# Molecular detection of *Leishmania infantum* and *Leishmania tropica* in rodent species from endemic cutaneous leishmaniasis areas in Morocco

**DOI:** 10.1186/s13071-017-2398-8

**Published:** 2017-10-02

**Authors:** Mohamed Echchakery, Carmen Chicharro, Samia Boussaa, Javier Nieto, Eugenia Carrillo, Ortega Sheila, Javier Moreno, Ali Boumezzough

**Affiliations:** 1 Ecology and the Environment Laboratory L2E, (URAC 32, CNRST ERACNERS 06), Faculty of Sciences Semlalia, Cadi Ayyad University, Marrakesh, Morocco; 2 WHO Collaborating Centre for Leishmaniasis, Parasitology Service, National Center of Microbiology Institute of Health Carlos III, Majadahonda, Madrid, Spain; 3 ISPITS-Higher Institute of Nursing and Technical Health Occupations, Ministry of Health, Marrakesh, Morocco

**Keywords:** *Leishmania tropica*, *L. infantum*, LnPCR, ITS1 PCR, Rodents, Morocco

## Abstract

**Background:**

Leishmaniasis remains a major public health problem in African nations, including Morocco, where little is known about the vertebrate reservoirs involved in the causal parasites’ transmission cycles. The present study investigates the role of rodent species as potential reservoirs of *Leishmania* spp. in central Morocco, where both *L. tropica* and *L. infantum* have been reported.

**Methods:**

Rodents were caught from 22 sites in central Morocco, by using Sherman metal traps, and identified morphologically. For each specimen, genomic DNA was extracted from different tissues using the Speed Tools DNA extraction Kit. Then, samples were PCR-analyzed, targeting the SSU rRNA gene to detect *Leishmania* spp. DNA, followed by amplification of the internal transcribed spacer 1 (ITS1) and its sequencing to identify the species.

**Results:**

A total of 197 rodents belonging to ten species were captured and identified: *Rattus rattus* (40.61%), *Mus musculus* (25.38%), *Apodemus sylvaticus* (8.63%), *Mus spretus* (7.11%), *Meriones shawi* (5.58%), *Rattus norvegicus* (4.57%), *Meriones libycus* (3.05%), *Mastomys erythroleucus* (2.03%), *Gerbillus campestris* (2.03%) and *Lemniscomys barbarus* (1.01%). Molecular analysis revealed the presence of *Leishmania* species in 18 specimens: six *R. rattus* (out of 80 captured; 7.5%), 11 *M. musculus* (out of 50 captured; 22%), and one *R. norvegicus* (out of 9 captured; 11.11%).

**Conclusions:**

To the best of our knowledge, *L. infantum* and *L. tropica* were identified in rodent species for the first time in Morocco. These findings suggest that rodent species may be involved in *L. infantum* and *L. tropica* transmission cycles in this country but that further studies are needed to confirm their role as reservoirs of *Leishmania* species in Morocco.

**Electronic supplementary material:**

The online version of this article (10.1186/s13071-017-2398-8) contains supplementary material, which is available to authorized users.

## Background

Leishmaniasis is a vector-borne infectious disease caused by members of the genus *Leishmania* (Kinetoplastida: Trypanosomatidae) and transmitted by phlebotomine sand flies (Diptera: Psychodidae) [1]. In the Mediterranean basin, the primary reservoir hosts of *Leishmania* spp. are wild mammals, mainly rodents and canids [2–5].

Leishmaniasis remains one of the major public health problems in Morocco where three *Leishmania* species coexist [6]. *Leishmania infantum* causes mainly zoonotic visceral leishmaniasis; *L. major* causes zoonotic cutaneous leishmaniasis and *L. tropica* causes anthroponotic cutaneous leishmaniasis [6]. Certainly, natural *Leishmania* infections have been reported in many rodent species, such as *Mus musculus*, *Rattus norvegicus*, *Rattus rattus* and *Apodemus sylvaticus* [7–17]. However, in Morocco, the only proven rodent reservoir of *Leishmania* (*L. major* MON 25) is *Meriones shawi* (Rodentia: Gerbillidae) [18, 19], despite the country being rich in rodent species [20].

The detection of infection in wild and domestic animals is the first step in identifying the different host reservoirs of *Leishmania* spp. Host incrimination depends on the accumulation of evidence based on five criteria [21, 22]: (i) geographical and temporal overlapping of vectors and hosts distributions; (ii) survival of the reservoir host long enough to permit transmission; (iii) higher infection prevalence; (iv) presence and frequency of parasites in the skin or the blood to be infective for the vector; and (v) detection of the same *Leishmania* species in human cases and the reservoir host.

In the present work, molecular methods were used to detect *Leishmania* infection in wild-caught rodents from central Morocco, from where both cutaneous and visceral leishmaniasis has been reported in humans [17, 23].

## Methods

### Study area

**Fig. 1:**
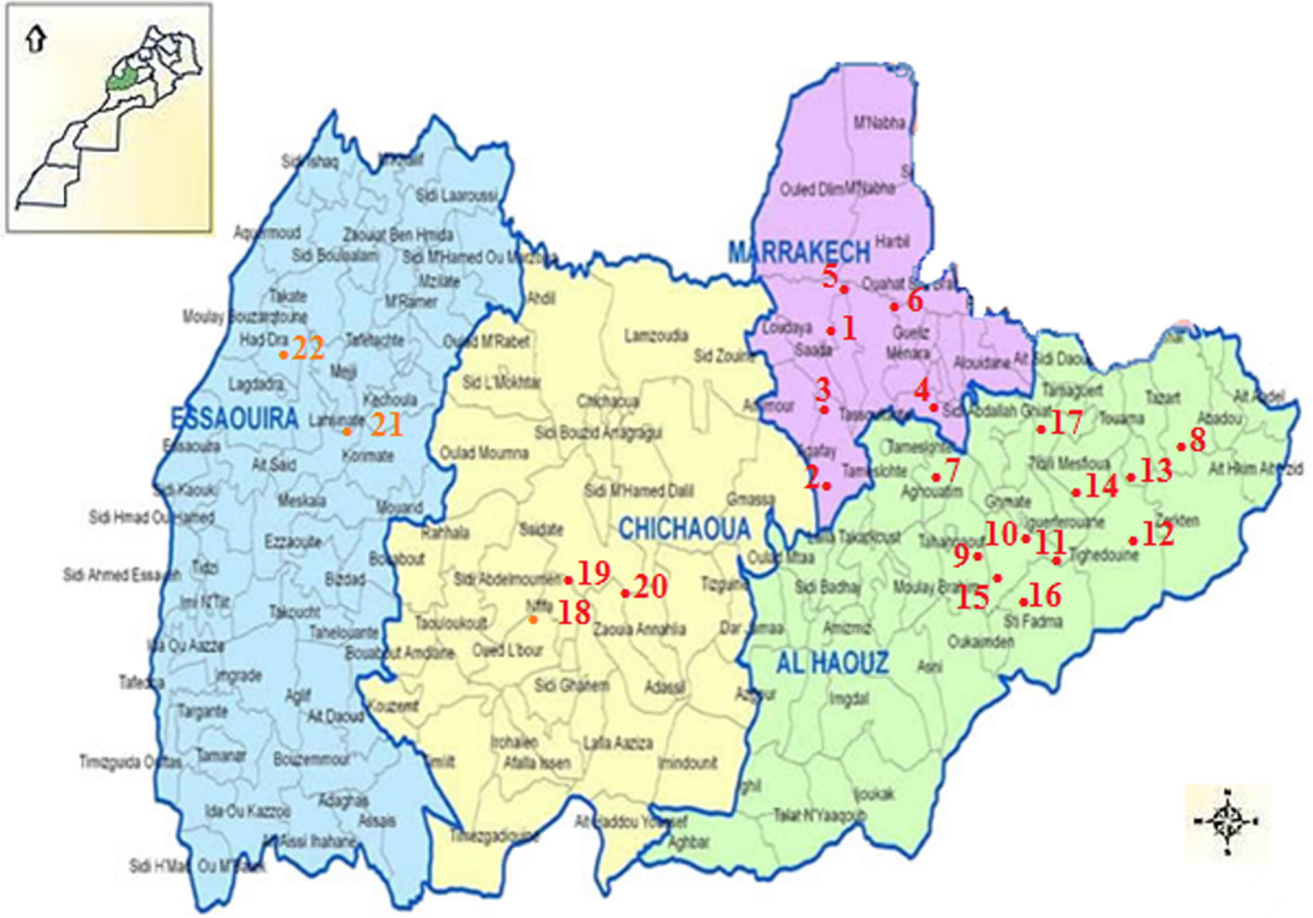
Local map showing the study area. Numbers (1–22) indicate sampling sites

This study was conducted in four regions of central Morocco: Al Haouz, Chichaoua, Essaouira and Marrakesh. A total of 22 sites (Fig. 1) with altitudes between 318 and 2579 m were sampled. Across the study area, the climate is arid to semi-arid on the plain (up to 450 m in Marrakesh) and humid in the Atlas Mountains (right up to 4167 m on Toubkal Mountain) and on the coast (up to 50 m in Essaouira).

### Rodent collection

The presence of rodents was surmised from cut plant stems and fresh droppings. Animals were captured using metal Sherman baited traps placed at the entrances of active burrows. For catching many rodent species, different baits were used in the same trap: bread with olive oil, tomatoes, potatoes and dates. The traps were set in the afternoon and recovered early the next morning. Forty traps were placed twice monthly (every 15 days) at each site between June 2014 and May 2015.

### Treatment of captured rodents and DNA extraction

**Fig. 2:**
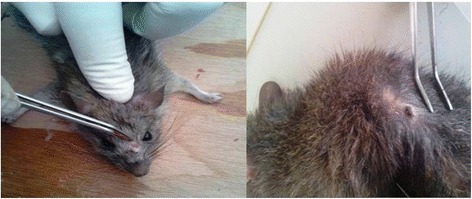
Skin lesions in rodent specimens

In the laboratory, caught rodents were anaesthetized with ether for the purposes of species identification and collecting data on weight, length (body and tail) and skin lesions (according to European decree NOR: AGRG1238767A 2013). Species identifications were made according to morphological characteristics [24]. Samples of liver, spleen, bone marrow and skin (ear lobe and, when available, skin lesions) (Fig. 2) were then harvested for parasite detection.25–30 mg of each tissue was removed with the aid of single-use forceps, scissors and scalpel blades, placed in sterile tubes, and stored at -20 °C for DNA extraction.

Genomic DNA was extracted using the Speed Tools DNA extraction Kit (Biotools, Madrid, Spain) following the manufacturer’s recommendations, and was eluted in a final volume of 200 μl of PCR-grade water. The extracts were stored at 4 °C until PCR analysis.

### Detection of *Leishmania* spp*.* DNA

The extracted DNA was screened for *Leishmania* spp*.* by the LnPCR amplifying a fragment from the small subunit ribosomal ribonucleic acid gene SSU rRNA [25, 26]. The first amplification step (Table 1) was performed using R221 (specific for order Kinetoplastida) and R332 primers (specific for the genera *Leishmania* and *Crithidia*) [25]. The PCR product was then tested in a subsequent amplification step with R233 and R333 primers of the genus *Leishmania*, according to the protocol developed by Van Eys et al. [25] and adapted and modified by Cruz et al. [27].

**Table 1:** PCR methods and primers sequences and main conditions used in this study

Gene	Primer	Product size (bp)	Primer sequence (5′-3′)	Amplification conditions	H_2_O (μl)^a^	MgCl_2_ (μl)	dNTP (0.2 mM) (μl)	Primer (μl)	Units *Taq* DNA polymerase (μl)	DNA (μl)	Total volume (μl)
SSU	R221	603	GGTTCCTTTCCTGATTTACG	94 °C for 5 min, 94 °C for 30s, 60 °C for 30s, 72 °C for 30s, 72 °C for 5 min (30 cycles)	30.6	5.0	1.0	1.0 (15 pmol)	1.4 (1 U/μl)	10	50
	R332		GGCCGGTAAAGGCCGAATAG								
	R223	358	TCCCATCGCAACCTCGGTT	94 °C for 5 min, 94 °C for 30s, 65 °C for 30s, 72 °C for 30s, 72 °C for 5 min (30 cycles)	10.6	2.5	0.5	0.5 (7.5 pmol)	0.7 (0.5 U/μl)	10^a^	25
	R333		AAAGCGGGCGCGGTGCTG								
								0. 25 (3.75 pmol)			
ITS1	LITSR	300–350	CTGGATCATTTTCCGATG	94 °C for 5 min, 94 °C for 30s, 53 °C for 30s, 72 °C for 30s, 72 °C for 5 min (30 cycles)	30.6	5.0	1.0	1.0 (15 pmol)	1.4 (1 U/μl)	10	50
	L5.8S		TGATACCACTTATCGCACTT								
	SAC	280–330	CATTTTCCGATGATTACACC	94 °C for 5 min, 94 °C for 30s, 57 °C for 30s, 72 °C for 30s, 72 °C for 5 min (30 cycles)	10.6	2.5	0.5	0.5 (7.5 pmol)	0.7 (0.5 U/μl)	10^b^	25
	VAN2		CGTTCTTCAACGAAATAGG								

^a^Sterile distilled water

^b^10 μl of a 1/40 dilution of the first PCR amplicons

A reamplification reaction was then performed in a 25 μl final volume, involving 10 μl of a 1/40 dilution of the first PCR amplicons as a template was added to a PCR mixture (Table 1). The components, total reaction volume and programs for the implementation of the LnPCR are detailed in Table 1. The amplification products were resolved on a 1.5% agarose gel, stained with Gel Red Nucleic Acid stain (Biotium, Fremont, California, USA), and visualized under UV light. Samples yielding a PCR product of 603 bp (Additional file 1: Figure S1) and 358 bp (Additional file 1: Figure S2), respectively, by first and second amplification reaction were deemed positive for *Leishmania* spp*.* DNA.

Negative controls without DNA were employed in all assays. DNA from the reference *Leishmania infantum* strain MHOM/FR/78/LEM75 was used as a positive control (Additional file 1: Figures S1-S3).

### Identification of *Leishmania* species

Samples shown to be positive by LnPCR were further analyzed to identify the *Leishmania* species by nested amplification of the ribosomal internal transcribed spacer 1 (ITS1-PCR).

The first PCR reaction was carried out using the primers LITSR/L5.8S as described by El Tai et al. [28] and Schönian et al. [29]. Amplification reactions were performed in volumes of 50 μl was added to a PCR mixture described in Table 1. For the second reaction, we used the primers SAC and VAN2 [30]. A reamplification reaction was then performed in a 25 μl final volume, involving 10 μl of a 1/40 dilution of the first PCR amplicons as a template was added to a PCR mixture described in Table 1.

Negative and positive controls were also included in this assay. All reactive were synthesized commercially (Biotools, B&M Labs, S.A., Madrid, Spain). All PCR products were visualized on 1.5% agarose gel as above (Additional file 1: Figure S3). Samples providing a 300–350 bp (Additional file 1: Figure S2) and 280–330 bp (Additional file 1: Figure S3), respectively, were deemed positive. The ITS1-PCR products were excised from the agarose gels and purified using the QIA quick extraction kit (Qiagen). They were then sequenced which allows to correctly identify all species of the genus *Leishmania* of the Old World [31], using the Big-Dye Terminator Cycle Sequencing Ready Reaction kit v3.1 and an automated ABI PRISM 377 DNA sequencer (Applied Biosystems, Foster City, CA, USA). These sequences were edited using BioEdit Sequence Alignment Editor Software v. 7.0.9.0 [32] and compared with others held in the GenBank database using BLAST software.

## Results

A total of 197 animals belonging to 10 rodent species were captured (Table 2): 80 *Rattus rattus* (40.61%), 50 *Mus musculus* (25.38%), 17 *Apodemus sylvaticus* (8.63%), 14 *Mus spretus* (7.11%), 11 *Meriones shawi* (5.58%)*,* 9 *Rattus norvegicus* (4.57%), 6 *Meriones libycus* (3.05%), 4 *Mastomys erythroleucus* (2.03%), 4 *Gerbillus campestris* (2.03%) and 2 *Lemniscomys barbarus* (1.01%).

**Table 2:** Rodent species captured in the study area

Species	Male	Female	Total	%
*Rattus rattus*	28	52	80	40.61
*Mus musculus*	14	36	50	25.38
*Apodemus sylvaticus*	6	11	17	8.63
*Mus spretus*	6	8	14	7.11
*Meriones shawi*	5	6	11	5.58
*Rattus norvegicus*	4	5	9	4.57
*Meriones libycus*	1	5	6	3.05
*Mastomys erythroleucus*	1	3	4	2.03
*Gerbillus campestris*	2	2	4	2.03
*Lemniscomys barbarus*	0	2	2	1.01
Total	67	130	197	100

*Leishmania* species was detected in 18 specimens (Table 3): 6 *R. rattus* (out of 80 captured; 7.5%), 11 *M. musculus* (out of 50 captured; 22%), and one *R. norvegicus* (out of 9 captured; 11.11%). Statistical analysis, using Chi-square test of independence, of infestation rate according to rodent species showed no significant correlation (*χ* ^2^ = 5.768, *df* = 2, *P* = 0.056).

**Table 3:** Molecular identification of *Leishmania* species from rodent species by tissue/organ

Rodent species and number of specimens	LnPCR				ITS1-PCR				*Leishmania* spp.	Identity (%)
	L	Sp	S	Sl	L	Sp	S	Sl		
*R. norvegicus* (*n* = 1/9)	P	P	N	–	P	P	N	–	*L. infantum*	99
*R. rattus* (*n* = 6/80)	P	P	N	–	P	P	N	–	*L. infantum*	99
	P	P	N	–	P	P	N	–	*L. infantum*	99
	N	N	P	P	N	N	P	P	*L. infantum*	99
	N	N	P	P	N	N	P	P	*L. infantum*	99
	N	N	P	–	N	N	P	–	*L. infantum*	98
	N	N	P	–	N	N	P	–	*L. infantum*	99
*M. musculus* (*n* = 9/50)	N	N	P	–	N	N	P	–	*L. infantum*	98
	N	N	P	–	N	N	P	–	*L. infantum*	99
	N	N	P	–	N	N	P	–	*L. infantum*	98
	N	N	P	–	N	N	P	–	*L. infantum*	99
	N	N	P	–	N	N	P	–	*L. infantum*	99
	N	N	P	–	N	N	P	–	*L. infantum*	100
	N	N	P	P	N	N	P	P	*L. infantum*	99
	N	N	P	P	N	N	P	P	*L. infantum*	99
	N	N	P	P	N	N	P	P	*L. infantum*	99
*M. musculus* (*n* = 2/50)	N	N	P	–	N	N	P	–	*L. tropica*	96
	N	N	P	–	N	N	P	–	*L. tropica*	97

*Abbreviations*: *L* Liver, *S* Skin, *Sl* Skin lesion, *Sp* Spleen, *N* Negative, *P* Positive, –, absence of skin lesion

*Leishmania infantum* DNA was detected in the different organs of 16 (8.12%) specimens (Table 3): in the liver (18.75%) and the spleen (18.75%) of three specimens (*n* = 3), and in the skin (81.25%) of 13 specimens (*n* = 13). *Leishmania tropica* DNA was found only in the skin of 2 (1%) specimens (Additional file 1: Figures S1-S3).

According to the region, *Leishmania* spp. DNA was detected in 3 of the 4 investigated regions: Al Haouz, Chichaoua and Essaouira (Table 4); with a very significant difference (*χ* ^2^ = 20.116, *df* = 3, *P* < 0.0001). *Leishmania infantum* was detected in the Al Haouz region in 14.28% (3/21) of *R. rattus*, and in 46.15% (6/13) of *M. musculus* and *L. tropica* was identified in Chichaoua region in 14.28% (1/7) of *M. musculus*.

**Table 4:** Rodent species infected with *Leishmania* species by region

	El Haouz		Chichaoua		Essaouira		Marrakesh	
	Neg	Pos	Neg	Pos	Neg	Pos	Neg	Pos
*R. rattus*	18	3 *L. infantum* (14.28%)	12	0	14	3 *L. infantum* (17.64%)	30	0
*M. musculus*	7	6 *L. infantum* (46.15%)	6	1 *L. tropica* (14.28%)	7	3 *L. infantum* (27.27%) + 1 *L. tropica* (9.10%)	19	0
*R. norvegicus*	0	0	0	0	2	1 *L .infantum* (33.33%)	6	0

*Abbreviations*: *Neg* Negative, *Pos* Positive

Both *Leishmania* species were detected in Essaouira region: *L. infantum* was identified in 17.64% (3/17) of *R. rattus*, in 33.33% (1/3) of *R. norvegicus*, and in 27.27% (3/11) of *M. musculus*; while, *L. tropica* was identified in 9.10% (1/11) of *M. musculus.* In Marrakesh region, no *Leishmania* spp*.* DNA was detected in any of the 55 animals captured (Table 4).

Ten animals showed ear lobe skin lesions (Fig. 2), of which 50% were confirmed positive for *L. infantum*. Three of these positive animals showed, in addition, splenomegaly and hepatomegaly (compatible with visceral leishmaniasis).

## Discussion

The identification of the natural hosts of *Leishmania* species is essential to understand the epidemiology of the disease. To our knowledge, there have been no data published since 1982 on natural *Leishmania* infection in rodent species in Morocco [19].

In the present study, *L. infantum* DNA was detected in six *R. rattus*, nine *M. musculus* (both anthropophilic species) and one *R. norvegicus*, while *L. tropica* DNA was detected in two *M. musculus.* This is the first time that *L. infantum* and *L. tropica* infections have been detected in rodent species in Morocco. In the wider Mediterranean region, *L. infantum* (which causes visceral and cutaneous zoonotic leishmaniasis) has been identified in naturally infected wild rodents including *R. rattus*, *R. norvegicus* and *M. musculus* [8, 10, 12, 15, 16, 33–35]. In Morocco, dogs make up the main host reservoir for *L. infantum* [17, 36]. However, on the Island of Montecristo (Italy), where no dogs are present, up to 15.5% of *R. rattus* individuals have been reported infected with *L. infantum* [37], maintaining a sylvatic transmission cycle.

*Leishmania tropica*-induced cutaneous leishmaniasis is commonly considered an anthroponotic disease that does not involve an animal reservoir [38], but zoonotic transmission has been demonstrated in Jordan, Palestine and Israel [39, 40].

In north Africa, several rodent species have been implicated in the transmission of *L. tropica* [39–42]: *Ctenodactylus gundi* has been proposed as a reservoir host in Tunisia [42, 43], while in Algeria, human cutaneous leishmaniasis caused by *Leishmania killicki* (a variant of *L. tropica*) is considered a zoonotic form with *P. sergenti* as a vector and *Massoutiera mzabi* as a reservoir [39]. However, in Morocco *L. tropica*is generally considered an anthroponotic species, despite its identification in dogs on many occasions [44–46].

In the present study, the regions of Chichaoua, Al Haouz and Essaouira were known to be endemic foci of *L. tropica-*induced cutaneous leishmaniasis for humans [47–49], while, *L. infantum* is reported responsible for sporadic human cases of visceral leishmaniasis in these areas [50, 51]. In the same study area, Boussaa et al. [23] reported *L. infantum-*induced leishmaniasis in dogs, which returned very high seroprevalence results (81.8 and 87.8% as determined by ELISA and Western blotting, respectively).

In addition, the vector sand flies *Phlebotomus perniciosus* and *P. sergenti* have been found engorged with rodent blood in southern Portugal [52] but also in central Morocco [53] where the sand fly composition is well established [54]. Svobodovà et al. [55] reported the transmission of *L. tropica* to mice by the bite of *Phlebotomus sergenti*, a species widespread in our study area [54].

The richness of rodent species (*n* = 10) across the present study area is favoured by ecological factors. Prevalence and abundance of rodent species areknown to be associated with abundant vegetation [56]. In arid and semi-arid regions, such in the study area, many authors suggested trophic cascade including precipitations, vegetation and rodent density 1 year later [57, 58].

## Conclusion

To the best of our knowledge, molecular detection of *L. infantum* DNA in *M. musculus*, *R. norvegicus* and *R. rattus*and of *L. tropica* DNA in *M. musculus* is reported here for the first time in Morocco. These results suggest the possible involvement of rodent species in *L. tropica* and *L. infantum* cycles. The present findings should be taken into consideration when developing programs to combat this disease in Morocco.

## Additional file

Additional file 1: Figure S1. Profile of agarose (1.5%) gel for diagnostic of *Leishmania* spp. in DNA extracted from tissue from rodent species, using LnPCR for to amplify part of the SSU rRNA gene for diagnosis. Lane MM: 100 bp molecular marker (DNA ladder); Lanes 1, 12: negative controls (no DNA PCR); Lane 13: positive control (MHOM/FR/78/LEM75); Lanes 2–11: DNA extracted of tissue from rodent species. LnPCR for first amplification was carried out using R221/R332 primers. **Figure S2.** Profile of agarose (1.5%) gel for diagnostic of *Leishmania* spp. in DNA extracted from tissue from rodent species, using LnPCR for to amplify part of the SSU rRNA gene for diagnosis. Lane MM: 100 bp molecular marker (DNA ladder); Lanes 1, 12, 14: negative controls (no DNA PCR); Lanes 13, 15: positive controls (MHOM/FR/78/LEM75); Lanes 2–11: DNA extracted of tissue from rodent species. LnPCR for second amplification was carried out using R233/R333 primers. **Figure S3.** Profile of agarose (1.5%) gel for characterization of *Leishmania* species in DNA extracted from tissue from rodent species, using ITS1-PCR for to amplify the ribosomal internal transcribed spacer 1 (ITS1) region. Lane MM: 100 bp molecular marker (DNA ladder); Lanes 1, 16, 18: negative controls (no DNA PCR); Lanes 17, 19: positive controls (MHOM/FR/78/LEM75); Lanes 2–15: DNA extracted of tissue from rodent species. ITS1-PCR for second amplification was carried out using SAC/VAN2 primers. (DOCX 314 kb)

## Abbreviations

ELISA: Enzyme-linked immunosorbent assay
ITS1: Internal transcribed spacer 1
LnPCR: *Leishmania* nested polymerase chain reaction
MM: Molecular marker
MON: *Leishmania major* Montpellier
SSU rRNA: Small subunit ribosomal ribonucleic acid
Tth: *Thermus thermophilus*
WHO: World Health Organization
