# Supplementary material for: Molecular detection of Leishmania infantum and Leishmania tropica in rodent species from endemic cutaneous leishmaniasis areas in Morocco
Source: Parasit Vectors. 2017 Oct 2;10:454. doi: 10.1186/s13071-017-2398-8 (PMC5625640; doi:10.1186/s13071-017-2398-8)
Supplement: Additional file 1: Figure S1. — Profile of agarose (1.5%) gel for diagnostic of Leishmania spp. in DNA extracted from tissue from rodent species, using LnPCR for to amplify part of the SSU rRNA gene for diagnosis. Lane MM: 100 bp molecular marker (DNA ladder); Lanes 1, 12: negative controls (no DNA PCR); Lane 13: positive control (MHOM/FR/78/LEM75); Lanes 2–11: DNA extracted of tissue from rodent species. LnPCR for first amplification was carried out using R221/R332 primers. Figure S2. Profile of agarose (1.5%) gel for diagnostic of Leishmania spp. in DNA extracted from tissue from rodent species, using LnPCR for to amplify part of the SSU rRNA gene for diagnosis. Lane MM: 100 bp molecular marker (DNA ladder); Lanes 1, 12, 14: negative controls (no DNA PCR); Lanes 13, 15: positive controls (MHOM/FR/78/LEM75); Lanes 2–11: DNA extracted of tissue from rodent species. LnPCR for second amplification was carried out using R233/R333 primers. Figure S3. Profile of agarose (1.5%) gel for characterization of Leishmania species in DNA extracted from tissue from rodent species, using ITS1-PCR for to amplify the ribosomal internal transcribed spacer 1 (ITS1) region. Lane MM: 100 bp molecular marker (DNA ladder); Lanes 1, 16, 18: negative controls (no DNA PCR); Lanes 17, 19: positive controls (MHOM/FR/78/LEM75); Lanes 2–15: DNA extracted of tissue from rodent species. ITS1-PCR for second amplification was carried out using SAC/VAN2 primers. (DOCX 314 kb) [file 13071_2017_2398_MOESM1_ESM.docx]

**Additional file 1**


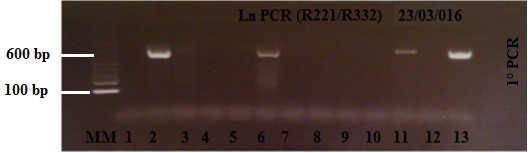


**Additional file 1: Figure S1.** Proﬁle of agarose (1.5%) gel for diagnostic of *Leishmania* spp. in DNA extracted from tissue from rodent species, using LnPCR for to amplify part of the SSU rRNA gene for diagnosis. Lane MM: 100 bp molecular marker (DNA ladder); Lanes 1, 12: negative controls (no DNA PCR); Lane 13: positive control (MHOM/FR/78/LEM75); Lanes 2–11: DNA extracted of tissue from rodent species. LnPCR for first amplification was carried out using R221/R332 primers.

**
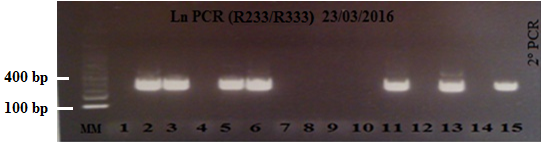
**

**Additional file 1: Figure S2.** Proﬁle of agarose (1.5%) gel for diagnostic of *Leishmania* spp. in DNA extracted from tissue from rodent species, using LnPCR for to amplify part of the SSU rRNA gene for diagnosis. Lane MM: 100 bp molecular marker (DNA ladder); Lanes 1, 12, 14: negative controls (no DNA PCR); Lanes 13, 15: positive controls (MHOM/FR/78/LEM75); Lanes 2–11: DNA extracted of tissue from rodent species. LnPCR for second amplification was carried out using R233/R333 primers.


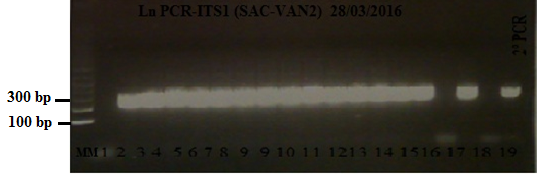


**Additional file 1: Figure S3.** Proﬁle of agarose (1.5%) gel for characterization of *Leishmania* species in DNA extracted from tissue from rodent species, using ITS1-PCR for to amplify the ribosomal internal transcribed spacer 1 (ITS1) region. Lane MM: 100 bp molecular marker (DNA ladder); Lanes 1, 16, 18: negative controls (no DNA PCR); Lanes 17, 19: positive controls (MHOM/FR/78/LEM75); Lanes 2–15: DNA extracted of tissue from rodent species. ITS1-PCR for second amplification was carried out using SAC/VAN2 primers.
